# ADVERSE CHILDHOOD EXPERIENCES AND DEPRESSIVE SYMPTOMS AMONG RACIALLY/ETHNICALLY DIVERSE OLDER ADULTS IN THE US

**DOI:** 10.1093/geroni/igac059.1906

**Published:** 2022-12-20

**Authors:** David Camacho, Julia Vazquez, Laura Vargas, Charles Henderson, Brenda J Jones-Harden

**Affiliations:** University of Maryland, Baltimore, Norwalk, California, United States; University of Maryland, Baltimore, Baltimore, Maryland, United States; University of Colorado School of Medicine, Department of Psychiatry, Aurora, Colorado, United States; Cornell University, Ithaca, New York, United States; University of Maryland, Baltimore, Baltimore, Maryland, United States

## Abstract

Adverse childhood experiences (ACEs) and depression are major public health concerns. However, few studies have examined the relationship between ACEs and mid- and late-life depression among racially/ethnically diverse groups. We explore this relationship among U.S. racially/ethnically diverse community-dwelling midlife and older adults (≥50 years of age). Guided by ACEs and Minority Stress Frameworks, we used general linear models to examine this relationship with data from Wave 3 of the National Social Life, Health, and Aging Project. We created an ACEs composite ranging from 0 to 7 (e.g., violence, health, poverty) and assessed the role of individual ACEs on depressive symptoms (CES-D). Final adjusted models (n:1424) included key demographic, health (e.g., chronic disease), social (living alone, social isolation, loneliness), and minority stress factors (e.g., limited access to healthcare and treatment, perceived discrimination). Results indicated that higher composite score ACEs (particularly childhood violence and poor health) were positively associated with higher levels of depressive symptoms. We found no interactions between race/ethnicity and ACEs. Our results suggest that ACEs contribute to the presence and severity of depressive symptoms into mid- and late-life adulthood. Consistent with Minority Stress Framework, common life-course stressors for minoritized groups may explain a lack of significant interactions in our models. Future research should explore the association of ACEs and other important health outcomes in diverse midlife and older adults. Finally, research is needed to examine if and how culturally appropriate depression interventions can be adapted to address the role of ACEs in later life health.

